# Tamoxifen Citrate Encapsulated Sustained Release Liposomes: Preparation and Evaluation of Physicochemical Properties

**DOI:** 10.3797/scipharm.0911-11

**Published:** 2010-07-12

**Authors:** Buddhadev Layek, Biswajit Mukherjee

**Affiliations:** 1 Department of Pharmaceutical Technology, Division of Pharmaceutics, Jadavpur University, Kolkata-700032, India; 2 Pharmaceutical Science Department, 1401 Albrecht Blvd, 123 Sudro Hall, North Dakota State University, Fargo, ND-58102, USA

**Keywords:** Tamoxifen citrate, Liposome, Soya phosphatidylcholine, Cholesterol

## Abstract

The present study was designed for the development of a stable sustained release liposomal drug delivery system for tamoxifen citrate (TC) using soya phosphatidylcholine (SPC), cholesterol (CH) and span 20 as main ingredients. Liposomes were prepared by formation of thin lipid film followed by hydration. The mean vesicle diameter was found to be 203.5 ± 19.5 nm with 21% of the liposomal population having average diameter below 76.72 ± 6.7 nm. There was a good vesicular distribution with the polydispersity index of 0.442 ± 0.03. The maximum loading of drug was determined to be 53.60% of the initial amount that is 34.58 μg of drug per mg of lipid. Amongst the different storage conditions, liposomes stored at 2–8°C were found to be most stable and only 4% of the drug was lost over the storage period of 5 weeks. In vitro release studies of liposomes showed that 50% of drug was released within 3 hours (h) whereas 95% drug was released in 30 h. This indicates the usefulness of the liposomal delivery system for sustaining the in vitro release of tamoxifen citrate.

## Introduction

Tamoxifen citrate, an anti-estrogen compound, is the first choice hormonal treatment of breast cancer in both pre- and post-menopausal women for last few decades. It is often used as an adjuvant therapy following primary treatment of early stage breast cancer [1–3]. Depending upon the dose and tissue, tamoxifen can act either as an anti-estrogenic or as an estrogen. To the breast, it is an anti-estrogenic, however it also has estrogenic effects on uterus and one of the significant side effects of postmenopausal tamoxifen treatment appears to be endometrial carcinoma [4]. Other side effects include liver cancer, venous thrombosis, pulmonary emboli and ocular effects such as retinopathy and corneal opacities [5]. These side effects were reported to be dose and concentration dependent [6]. Therefore the aim of tamoxifen therapy for long term chemoprevention of breast cancer is to deliver a lower dose of drug over a prolong period of time.

In the present investigation, our main objective was to develop a simple vesicular delivery system for tamoxifen citrate which can deliver drug at a lower concentration over a prolong period of time and thereby reducing the potential dose related side effects.

## Results and Discussion

To determine the drug-excipient interactions FTIR studies were conducted before development of the formulations. Drug-excipient interaction study is one of the most important parameters, which depicts much information regarding the stability of formulations, drug release from them and drug availability patterns [7].

**Fig.1.**
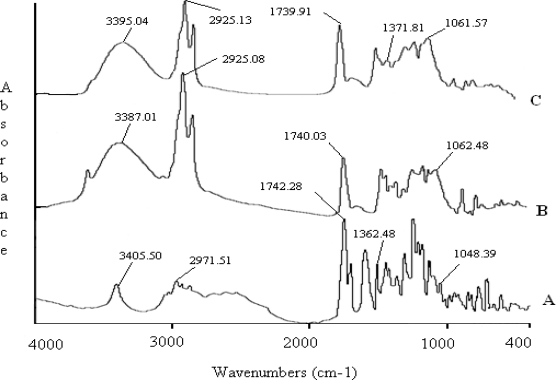
FTIR spectrums: (A) Pure drug TC; (B) Physical mixture of excipients; (C) Physical mixture of drug and excipients

Fig. 1C shows the minor shifting of some peaks compared to pure drug (Fig. 1A) and physical mixture of excipients (Fig. 1B), like aliphatic alcoholic O-H stretch (3405.50– 3395.04 cm^−1^), phenolic C-O stretch (1362.48–1371.81 cm^−1^), and C-O stretch of ether (1048.39–1061.57 cm^−1^). These shifts may be because of formation of hydrogen bonds, or some other weak forces like van der Wall forces or dipole moment amongst the polar functional groups of drug and phospholipids moieties. However, these interactions may favor the formation of vesicular shape, stability of structure and slower drug release [8].

In order to determine the influence of formulation components on the percent drug loading (PDL), composition with varying ratios of drug, SPC and CH were studied (data show mean ± S.D. and n = 3) (Table 1). For CH free SPC liposomes (TCL 1-TCL 4), maximum drug loading of 43.83% could be achieved at a drug-lipid ratio of 1:10 (w/w). The drug entrapment value can be further increased by 4–28% with the addition of 10 to 60% (w/w) of CH (TCL 5-TCL 10, Table 1). Results showed that incorporation of CH enhanced the percent of drug entrapment up to 55% (w/w) but further increase in CH decreases this value. The addition of CH has been shown to lend greater stability to the bilayer membranes by raising the gel liquid transition temperature of the vesicles. This stability decreases leakage of the vesicle and stabilizes it against osmotic gradient and thereby increasing the drug loading [9]. The further increase in the amount of CH reduces the drug entrapment because CH itself is incapable to form vesicles and also hinder the entry of drug molecule by occupying the space in phospholipid bilayer.

**Fig. 2.**
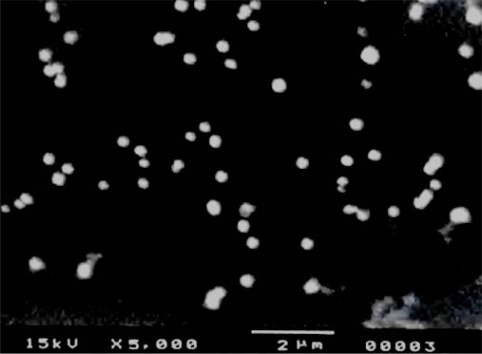
SEM photograph of drug loaded liposomes (TCL 9)

The size range of TCL 9 blank liposomes (without drug) was found to be within 50 to 800 nm with 46.86% of the liposomal population having average diameter of 153.2 ± 10.2 nm. The mean vesicle diameter was found to be 205 ± 15.2 nm (Table 2). The size range of TCL 9 drug loaded liposomes (Fig. 2) was found to be 38 to 700 nm, with 21.01% of the liposomal population having average size of 76.72 ± 6.7 nm. The mean vesicle diameter was found to be 203.5 ± 19.5 nm (Table 2).

Results showed that there was no significant difference between the size of blank and drug loaded liposomes. This may be attributed to the fact that TC is encapsulated in the lipid membrane [10]. The reproducibility of the liposomal formulation with respect to size was confirmed by preparing the formulation three times.

When zeta potential was studied to understand the surface charges of the vesicles it was noticed that zeta potential of the formulation without drug was strongly negative, whereas those with drug became less negative (Table 2). This is due to the cationic charge present on the drug having neutralized the surface charges existed on the formulation surface [11]. Although, the zeta potential of drug loaded liposomes decreased but still was sufficient to prevent the fusion of vesicles as the colloids with high zeta potentials (positive or negative) are electrically stabilized, while colloids with low zeta potentials tend to coagulate or flocculate.

**Fig. 3.**
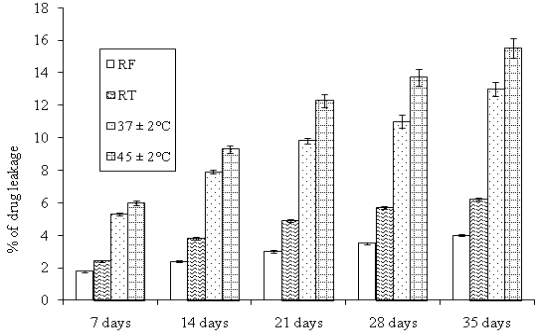
Extent of drug leakage from liposomes (TCL 9) at different storage conditions. Data shows mean ± S.D. (n=3)

The storage stability of TCL 9 liposomes were investigated in terms of aggregation, fusion and vesicle disruption tendencies. The bar diagram (data show mean ± S.D. and n = 3) (Fig. 3) shows the leakage of drug from liposomes at different storage conditions over the period of 5 weeks. Furthermore, it was found that approximately 13–15.5% of drug was lost at elevated temperatures of 37 ± 2°C and 45 ± 2°C, respectively. On the other hand almost 93.8 and 96% of drug remained incorporated at room temperature (RT) and refrigeration temperature (RF), respectively which are in accordance with the reported results [12]. TCL 9 liposomes exhibited excellent stability in terms of aggregation, fusion and vesicle disruption. This high stability of liposomes may be due to the cementing effect of cholesterol and thereby preventing the leakage of drug from vesicles during storage. The leakage of drug from the vesicles stored at elevated temperature might be due to the effect of high temperature on the gel to liquid transition of lipid bilayers together with possible chemical degradation of the phospholipid component [12]. Therefore this study suggests the storage of liposomal formulation in refrigerator in order to prevent loss of drug from the liposomes.

**Fig. 4.**
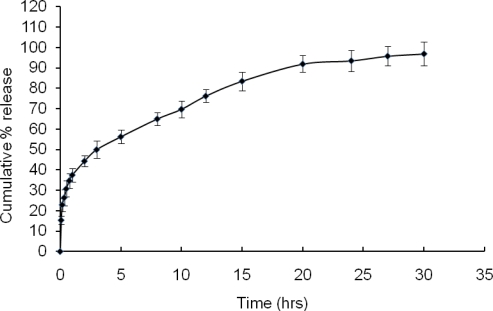
In vitro release of TC from the liposomes (TCL 9) using PBS buffer (pH 7.4) at 37°C. Data shows mean ± S.D. (n=3)

During in vitro release studies of tamoxifen from the liposomes, it was found that about 50% (Fig. 4) of drug was released within 3 h and about 95% of drug was released after 30 h. This extended release could be attributed to the decreased membrane fluidity of phospholipid bilayer by cholesterol and tamoxifen itself [13] and thereby preventing the burst release [14] as well as decrease the rate of drug released from vesicles. Therefore, the optimized formulation was able to release the drug over a prolonged period of time to reduce the dose related toxic effects.

## Experimental

### Materials

Tamoxifen citrate (TC) was a kind gift from Biochem Pharmaceutical Industries, India. Soya phosphatidylcholine (SPC) was purchased from HiMedia Laboratories Pvt. Ltd, Mumbai, India. Cholesterol (CH) was procured from E. Merck (India) Ltd., Mumbai, India. All other ingredients used in the study were of analytical grade.

### Methods

Liposomes were prepared by thin film hydration method. The weighed amounts of TC, SPC, CH, span 20 and BHT (2 % w/w of total lipid) (Table 1) were dissolved in chloroform-methanol mixture (2:1, v/v). The mixture was placed in a rotary vacuum evaporator with an aspirator and was rotated at 120 rpm. The temperature of the water bath was maintained at 35–40°C to evaporate the solvent. Subsequently, the flask was kept in a vacuum desiccator overnight for complete removal of residual solvent. The dried lipid film was hydrated with phosphate buffer solution (PBS, pH 7.4) in a rotary vacuum evaporator maintained at 60°C and rotated at 120 rpm until the lipid film was dispersed in the aqueous phase. The size reduction of vesicles was done by a bath-sonicator (30 ± 2 KHz) at 60°C for 1 h. Then the dispersion was left undisturbed at room temperature for 1–2 h for complete vesicle formation followed by storage at 4°C in an inert atmosphere for 24 h. Next day the preparation was centrifuged at 5000 rpm at 4°C for 5 min. The supernatant containing the vesicles in each case was taken for further studies.

#### Fourier transform, infrared (FTIR) study

The pure drug TC, physical mixture of SPC, CH and BHT, and physical mixture of TC, SPC, CH and BHT were mixed separately with infrared (IR) grade KBr in the ratio of 1:100 and corresponding pellets were prepared by applying 15000 lb of pressure in a hydraulic press. The pellets were scanned in an inert atmosphere over a wave number range of 4000-400 cm^−1^ in Magna IR 750 series II (Nicolet, USA) FTIR instrument.

#### Size distribution and zeta potential

Different batches of liposomes were monitored for their morphological attributes with the help of scanning electron microscope (SEM) (JSM, JEOL, Tokyo, Japan). Fusion and crystallization were detected by optical microscopy. Size distribution and Zeta potential of liposomes were measured by dynamic light scattering method using Zetasizer nano ZS (Malvern Instruments Ltd., UK) in triplicate.

#### Drug loading study

Freshly prepared liposomal suspensions (with or without drug) were ultracentrifuged at 120000 x g at 25°C for 1 hr (Sorvall, USA) with fix rotor type s 20/20 (DuPont, USA) to pelletize liposomes [15]. The pellet was then lyophilized and resulting powder containing liposomes was dissolved in ethanol to obtain a clear solution and absorbance was measured at 274 nm using UV/VIS spectrophotometer (Beckman, USA). Liposomes prepared without drug were treated in similar way and used as blank for the above study. The drug content was then determined from the standard curve. The percent drug loading for the prepared liposomes was calculated as in Eq. 1.

Eq. 1. $$\%\;\text{Drug}\;\text{loading}\;=\frac{\text{Entrapped}\;\text{drug}\;\text{(mg)}}{\text{Total}\;\text{drug}\;\text{added}\;\text{(mg)}}\cdot 100$$

#### Storage stability studies

The drug retentive capacity of the vesicles was studied by keeping the liposomal dispersion at four different temperatures i.e., 2–8°C (RF), 25 ± 2°C (RT), 37 ± 2°C and 45 ± 2°C for a period of 5 weeks [16] in sealed ampoules after flushing with nitrogen. The drug content of vesicles was determined periodically.

#### In vitro drug release

In vitro release of TC from liposomes was conducted by dialysis in a dialysis sac (Sigma, 12000 MW cut off) with 200 ml of PBS (pH 7.4) at 37°C [17]. Briefly, in a 250 ml conical flask, 200 ml of PBS was taken. One ml of formulation was taken into a dialysis bag and dipped into the buffer solution. The flask was kept on a magnetic stirrer. Stirring was maintained at 250 rpm and the temperature of the buffer was maintained at 37°C. Sampling was done by withdrawing 0.5 mL from the released medium with the help of micropipette and 0.5 mL of fresh buffer was added. Samples were analyzed using a spectrophotometer at a wave length of 274 nm. With the help of the standard curve prepared earlier, drug concentration was measured.

## Figures and Tables

**Tab. 1.** Effect of drug-lipid ratio and amount of cholesterol on TC loading in SPC liposomes

**Formulation code**	**Composition in ratio TC:SPC:CH (weight ratio)**	**Drug entrapped (mg) (mean±S.D., n = 3)**	**Percent drug loading**	**Liposome drug-lipid ratio (μg of drug : mg of lipid)^a^**
TCL 1	5:100:0	1.99±0.13	39.80	19.9:1
TCL 2	7.5:100:0	3.17±0.14	42.26	31.7:1
TCL 3	10:100:0	4.38±0.23	43.83	43.83:1
TCL 4	12.5:100:0	4.33±0.13	34.64	43.30:1
TCL 5	10:100:10	4.57±0.19	45.68	41.52:1
TCL 6	10:100:20	4.76±0.16	47.60	39.66:1
TCL 7	10:100:40	5.02±0.20	50.21	35.86:1
TCL 8	10:100:50	5.20±0.16	51.97	34.65:1
TCL 9	10:100:55	5.36±0.18	53.60	34.58:1
TCL 10	10:100:60	5.07±0.17	50.72	31.70:1

TCL…tamoxifen citrate liposomes; TC…tamoxifen citrate, SPC…soya phosphatidylcholine, CH…cholesterol;

a ratio was determined based on the initial amount of lipid used

**Tab. 2.** Size distribution and zeta potential of blank and drug loaded liposomes

**Sample name**	**Measurement temperature (°C)**	**Average particle size (nm) (n = 3)**	**Polydispersity index (n = 3)**	**Zeta potential (mV) (n = 3)**
TCL 9_blank_	25	205.0 ± 15.2	0.351 ± 0.02	−56.09 ± 4.3
TCL 9_drug_	25	203.5 ± 19.5	0.442 ± 0.03	−36.88 ± 3.8
